# Perspectives of key stakeholders regarding task shifting of care for HIV patients in Mozambique: a qualitative interview-based study with Ministry of Health leaders, clinicians, and donors

**DOI:** 10.1186/s12960-015-0009-3

**Published:** 2015-04-01

**Authors:** Alison S Rustagi, Rosa Marlene Manjate, Stephen Gloyd, Grace John-Stewart, Mark Micek, Sarah Gimbel, Kenneth Sherr

**Affiliations:** Department of Global Health, Schools of Public Health and Medicine, University of Washington, Harris Hydraulics Building, 1510 NE San Juan Road, Box 357965, Seattle, WA 98195-7765 USA; Ministry of Health, Maputo, Mozambique; Health Alliance International, Seattle, WA USA; Department of Allergy and Infectious Diseases, School of Medicine, University of Washington, Seattle, WA USA; Department of Pediatrics, School of Medicine, University of Washington, Seattle, WA USA; Department of Family and Child Nursing, School of Nursing, University of Washington, Seattle, WA USA

**Keywords:** Task shifting, HIV/AIDS, Mozambique, Antiretroviral therapy

## Abstract

**Background:**

Task shifting is a common strategy to deliver antiretroviral therapy (ART) in resource-limited settings and is safe and effective if implemented appropriately. Consensus among stakeholders is necessary to formulate clear national policies that maintain high-quality care. We sought to understand key stakeholders’ opinions regarding task shifting of HIV care in Mozambique and to characterize which specific tasks stakeholders considered appropriate for specific cadres of health workers.

**Methods:**

National and provincial Ministry of Health leaders, representatives from donor and non-governmental organizations (NGOs), and clinicians providing HIV care were intentionally selected to represent diverse viewpoints. Using open- and closed-ended questions, interviewees were asked about their general support of task shifting, its potential advantages and disadvantages, and whether each of seven cadres of non-physician health workers should perform each of eight tasks related to ART provision. Responses were tallied overall and stratified by current job category. Interviews were conducted between November 2007 and June 2008.

**Results:**

Of 62 stakeholders interviewed, 44% held leadership positions in the Ministry of Health, 44% were clinicians providing HIV care, and 13% were donors or employed by NGOs; 89% held a medical degree. Stakeholders were highly supportive of physician assistants performing simple ART-related tasks and unanimous in opposing community health workers providing any ART-related services. The most commonly cited motives to implement task shifting were to increase ART access, decrease physician workload, and decrease patient wait time, whereas chief concerns included reduced quality of care and poor training and supervision. Support for task shifting was higher among clinicians than policy and programme leaders for three specific task/cadre combinations: general mid-level nurses to initiate ART in adults (supported by 75% of clinicians vs. 41% of non-clinicians) and in pregnant women (75% vs. 34%, respectively) and physician assistants to change ART regimens in adults (43% vs. 24%, respectively).

**Conclusions:**

Stakeholders agreed on some ART-related task delegation to lower health worker cadres. Clinicians were more likely to support task shifting than policy and programme leaders, perhaps motivated by their front-line experiences. Harmonizing policy and programme managers’ views with those of clinicians will be important to formulate and implement clear policy.

**Electronic supplementary material:**

The online version of this article (doi:10.1186/s12960-015-0009-3) contains supplementary material, which is available to authorized users.

## Background

Worldwide, an estimated 35 million people are currently living with HIV, and of those, nearly 14 million need antiretroviral therapy (ART) but are untreated [1]. Current numbers of health workers are inadequate to provide ART to all eligible patients, particularly in settings with high HIV burdens. Task shifting of ART-related care from higher to lower levels of health workers has been used as a solution to expand ART access by increasing the overall number of ART providers [2,3]. Task shifting is effective and safe if properly implemented; a recent Cochrane review found that nurses or physician assistants who are adequately trained and supported can initiate ART and follow ART patients without compromising the quality of care [4], corroborating the results of prior research [5,6].

Mozambique has been a leader in task shifting, notably with the training of mid-level surgical technicians [7]. Like other sub-Saharan African countries, Mozambique has adopted task shifting of ART-related care to address its high HIV disease burden. Mozambique ranks among the lowest in the world in terms of trained human resources for health capacity, with only 0.416 physicians, nurses, and midwives per 1000 people [8], far below the World Health Organization (WHO)’s minimum threshold of 2.28 per 1000 people [2]. The Mozambican health system has four tiers of training among cadres of non-physician health workers: (1) superior-level providers, who receive at minimum 4 to 5 years of training, (2) mid-level providers, who receive approximately two and a half years of training, including physician assistants (*técnicos de medicina*), maternal and child health mid-level nurses, (*enfermeiras de saúde materna e infantil nível médio*), and general mid-level nurses (*enfermeiro geral nível médio*); (3) basic-level providers, who receive approximately one and a half years of training, including medical technicians (*agentes de medicina*), maternal and child health basic-level nurses (*enfermeiras saúde materna e infantil nível básico*), and general basic-level nurses (*enfermeiro geral nível básico*); and (4) elementary-level providers, a heterogeneous group including elementary-level nurses and midwives (which have been largely phased out), and community health workers, which are divided into a formal cadre that receives 6 months of training (*agentes polivalentes elementares*) and an informal cadre disease- or service-specific activists (*activistas comunitários*) who often receive 1 to 2 weeks of training. In 2006, the Ministry of Health in Mozambique began training physician assistants to initiate first-line ART and provide follow-up care to uncomplicated, non-pregnant, HIV-positive patients [9].

While many studies have quantified the impact of task shifting on health outcomes [4,5], the degree of support for task shifting of ART provision among stakeholders is not well defined. Support from national-level stakeholders is critical to implement task shifting in a safe, sustainable, and equitable manner and therefore maximize its benefits while minimizing the risk of harm [4]. Leadership and governance—which includes health sector policies, and collaboration and coalition building among stakeholders—is one of the WHO’s six core building blocks for health system strengthening and “arguably the most complex but critical” of the six [10]. Stakeholder support is crucial in the context of task shifting, as “any long-term success of task shifting strategies hinges on serious political and financial commitments” [11].

Front-line health workers appear to be generally supportive of task shifting. In a South African study, nurses and physicians had general support for ART-related task shifting, such as initiation of therapy or follow-up of patients on ART, when adequate training and supervision is provided [12]. However, this study was performed in the context of a randomized controlled trial, and consequently, its conclusions may not be broadly generalizable. In a qualitative study from Mozambique and Zambia, front-line health workers and health facility managers described task shifting as an everyday reality essential to provide basic care to an overwhelming volume of patients [13].

Few studies have reported national-level stakeholders’ opinions on task shifting of ART provision. In Uganda, a qualitative study with 34 individual interviews and eight focus groups that included policy-makers, health workers, and health service managers found generally supportive attitudes towards task shifting of HIV/AIDS services [14], though the authors did not quantify the number of respondents who supported or opposed task shifting, nor they stratify by stakeholder category. Another study from Uganda found that policy-makers and highly trained health professionals opposed the creation of any policy regarding task shifting and task shifting itself, despite the fact that it has been integral to the provision of medical care throughout the Ugandan health system for decades [15]. One case study from Malawi—focused on task shifting in general among community health workers—found that confusion about respondents’ assigned duties and lack of training to perform duties that supervisors delegated to them were sources of frustration and low quality of care [16]. The authors concluded that enhanced cooperation within the Ministry of Health (MoH) and between the MoH and other non-governmental organizations (NGOs), including multi- and bilateral agencies, was necessary to resolve this and other implementation issues [16]. Though data on national stakeholders’ view on task shifting are sparse, even less clearly defined is the variation in support that may exist between different categories of national stakeholders. Discordant views on task shifting of HIV care duties may impede its successful implementation and undermine attempts to formulate policy, as has been documented in malaria vector management in Mozambique [17].

A clear definition of which services are appropriate to shift to specific cadres is needed to articulate clear policies around shifting tasks such as ART initiation and follow-up care to less highly trained health workers and to deliver adequate training and supervision strategies to ensure high-quality health services. In the absence of explicit direction, front-line health workers may assume tasks for which they are inadequately trained, supported, or monitored [16]. While one qualitative study in Kenya reported stakeholders’ views on general health promotion, prevention, and curative tasks that could be shifted to lower cadre health workers (in that study, community health workers) [18], no studies to date have reported national-level leaders’ views on where to draw the line in terms of task shifting of ART-related care. A better understanding of stakeholder views on which cadres should have authority to perform ART-related tasks in specific patient populations would be useful in developing appropriate human resource policies, training materials, and support strategies.

The aim of this study was to describe the perspectives of key stakeholders regarding task shifting of ART provision. Additionally, we sought to define which tasks stakeholders deemed appropriate for different health worker cadres, different patient populations, and if there were any differences across stakeholder categories.

## Methods

Data were collected in semi-structured, one-on-one interviews that were conducted with individuals intentionally selected by two individuals (K.S. and a senior Ministry of Health manager) to represent the key stakeholders in the development of MoH policies for the provision of HIV care in Mozambique, including (1) MoH leadership at the national and provincial levels, (2) health sector representatives of donors and NGOs at the national level, and (3) HIV clinicians providing HIV care and treatment in six public hospitals that had pioneered ART delivery in Mozambique leading up to, and during, the initiation of the national HIV Care and Treatment Program (between 2002 and 2004). For the MoH leadership at a national level, we mapped out the key individuals based on leadership positions and/or programmatic relevance (i.e., heads of programmes germane to each topic). All but two of the recruited individuals consented to be interviewed and were interviewed. For MoH staff at a provincial level, we selected provinces from the north, south, and central regions (two provinces per region) and targeted the provincial chief medical officer and head of the HIV programme; those present on the day of the interviews were interviewed. For hospital staff, we targeted MoH-run health facilities providing ART and selected purposively to overlap with the provinces we were visiting to interview MoH provincial staff. We attempted to interview clinical leadership and a non-random sample of clinical staff in these facilities based on availability, until saturation was reached. For leaders of NGOs and donors, we listed the key donors and NGOs in Mozambique at the time and reached out to the individuals involved in policy development, support for public HIV care, or provision of HIV care (one individual per organization). There were no exclusion criteria for any of the groups.

The study instrument (Additional file 1) was developed to include both closed- and open-ended questions, with the intent of characterizing respondents’ perceptions regarding task shifting in general and regarding the shifting of specific ART-related tasks to lower-level health cadres. Interview questions were tested for understandability and contextual appropriateness in a small number of individuals (representing national and provincial leadership, NGO/donors, and clinicians, reflecting similar backgrounds to respondents) before interviewing respondents. All interview questions were developed in Portuguese, all respondents spoke Portuguese, and all interviews were conducted in Portuguese. Interviews were not recorded. All interviews were conducted in the private offices of interviewees or in private spaces in HIV clinics. Notes were taken in Portuguese by the data collection team. The data collectors recorded responses to open-ended questions as summary statements. If the question or answer was not clear, the interviewers repeated the question, or repeated their interpretation of the respondent’s answer, to ensure that the message was mutually understood.

Interviewers collected data on interviewees’ demographics, education, and employment using closed-ended questions. Attitudes about the delegation of tasks to different levels of health-care workers were ascertained using both closed- and open-ended questions. For each of seven cadres of health workers in Mozambique (Table 1), interviewees were asked whether the given cadre should perform each of eight tasks related to the provision of ART (yes/no): initiate ART in adults, pregnant women, adults with tuberculosis, or children; follow adults or children after ART initiation; change ART regimen in adults or children. These tasks were chosen based on our experience, because they would be more representative of key steps to expanding and sustaining ART coverage. In presenting results, we ordered tasks from simple (e.g., following patients in whom ART has already been initiated) to complex (e.g., changing ART regimen in a child), based on the proportion of participants who thought that a given task would be appropriate to delegate to lower cadre health workers. Finally, interviewees were asked to give up to three benefits and up to three risks of delegating ART tasks to physician assistants and up to three suggestions to ensure the quality of services provided by non-physician health workers. All interviews were conducted by one of three people and took place in person between November 2007 and June 2008.

**Table 1:** **Relevant cadres of non-physician health workers in Mozambique, by training level**

**Level**	**Title (Portuguese)**	**Title (English)**	**Training**
Superior	*Médico*	Physician	6–7 years
Mid-level	*Técnico de medicina*	Physician assistant	2.5 years
	*Enfermeira SMI nível médio*	MCH nurse (mid-level)	2.5 years
	*Enfermeira nível médio*	General nurse (mid-level)	2.5 years
Basic level	*Agente de medicina*	Medical assistant	1.5 years
	*Enfermeira SMI nível básico*	MCH nurse (basic level)	1.7 years
	*Enfermeira nível básico*	General nurse (basic level)	1.5 years
Elementary	*Agentes polivalentes elementares*	Community health worker	6 months
n/a^a^	*Activistas comunitários*	Disease- or service-specific community health worker	1–2 weeks

Abbreviations: SMI, *saúde maternal e infantil*; MCH, maternal and child health.

^a^Informal cadre.

The frequencies of responses to closed-ended questions were tabulated overall and by employment of the respondent (policy-maker/clinician/donor or NGO worker) using simple percentages. Tables presenting the percentage of respondents who thought that a given health worker should perform a given task were colour-coded to highlight the direction and magnitude of agreement among respondents. Ranges for the colour-coding were chosen to highlight near-universal support (≥90% in darkest green) or opposition (<10% in darkest red) of the use of specific cadres to perform specific tasks; the width of the ranges was slightly larger for middle-level agreement (e.g. 40% to <60% was colour-coded white). Open-ended answers were analysed thematically to identify the most commonly cited responses. This content analysis focused on the most common answers, though all responses were tabulated and included in the results. We chose not to report *p* values because the goal of this study was not to test a hypothesis or to draw inferences beyond the study sample, but rather to describe major themes in the attitudes of stakeholders. All analyses were conducted using Stata, version 13.1 (College Station, TX).

This study was approved by the Institutional Review Boards at the University of Washington and the Ministry of Health of Mozambique. All respondents provided written consent to study participation prior to being interviewed.

## Results

### Demographics of individuals interviewed

Sixty-four individuals were selected and 62 agreed to be interviewed. Of the 62 interviewees, 44% held leadership positions within the MoH (including national directors and deputy directors, programme managers, provincial directors, and provincial chief medical officers), 44% were working as clinicians providing HIV care at tertiary or quaternary public hospitals, and 13% were multilateral or bilateral donors or employed by NGOs (Table 2). Most MoH leaders (71%) and donors/NGO workers (75%) lived in Mozambique’s capital city of Maputo, whereas most clinicians (95%) lived outside the capital. Physicians made up the majority of interviewees (89% of MoH leaders, 52% of clinicians, 63% of donor/NGO workers). Donor/NGO workers were more likely to be female (63%) than MoH leaders (48%) or clinicians (26%). Most (71%) interviewees had over a decade of work experience.

**Table 2:** **Characteristics of interviewees by current employment category (** ***n*** **= 62)**

**Characteristic**	**Policy-maker**		**HIV care provider**		**Donor/NGO**	
	**(** ***n*** **= 27)**		**(** ***n*** **= 27)**		**(** ***n*** **= 8)**	
Female, *n* (%)	13	(48.2)	7	(25.9)	5	(62.5)
Age, mean (SD)	42	(6.8)	39.5	(10.3)	43.3	(5.3)
Reside in capital city, *n* (%)	17	(70.8)	1	(4.2)	6	(75.0)
Training, *n* (%)						
Non-specialized MD	10	(37.0)	10	(37.0)	0	0
Specialized MD	14	(51.9)	4	(14.8)	5	(62.5)
Medical officer	1	(3.7)	13	(48.2)	0	0
Other	2	(7.4)	0	(0)	3	(37.5)
Years of experience, mean (SD)	15.2	(7.5)	11.9	(8.9)	16.5	(7.3)
Current job includes clinical duties, mean (SD)	1	(4.0)	27	(100)	1	(13.0)

### Tasks that specific cadres of health workers should be able to perform

**Figure 1:**
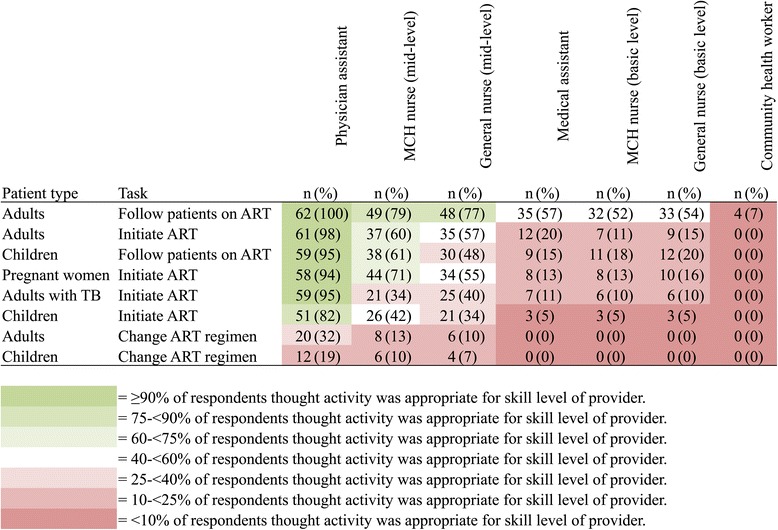
**Support for use of specific health worker cadres to perform specific ART-related tasks (** ***n*** **= 62 interviewees).**

Overall, more than 90% of interviewees thought that physician assistants (*técnicos de medicina*) should be able to initiate ART in adults with and without TB and in pregnant women and to provide follow-up care to adults and children who are already on ART (Figure 1). Eighty-two percent of interviewees thought that physician assistants should be able to initiate ART in children. Interviewees were almost unanimous that community health workers should not provide any of the eight ART-related tasks included in the study instrument and that basic-level health workers should not initiate ART in children or change ART regimens in children or adults. There was a less consensus on delegating moderately complex ART-related tasks to mid-level cadres of health workers; for example, 71% and 55% of interviewees expressed that mid-level maternal and child health (MCH) nurses and general mid-level nurses, respectively, should be able to initiate ART in pregnant women.

**Figure 2:**
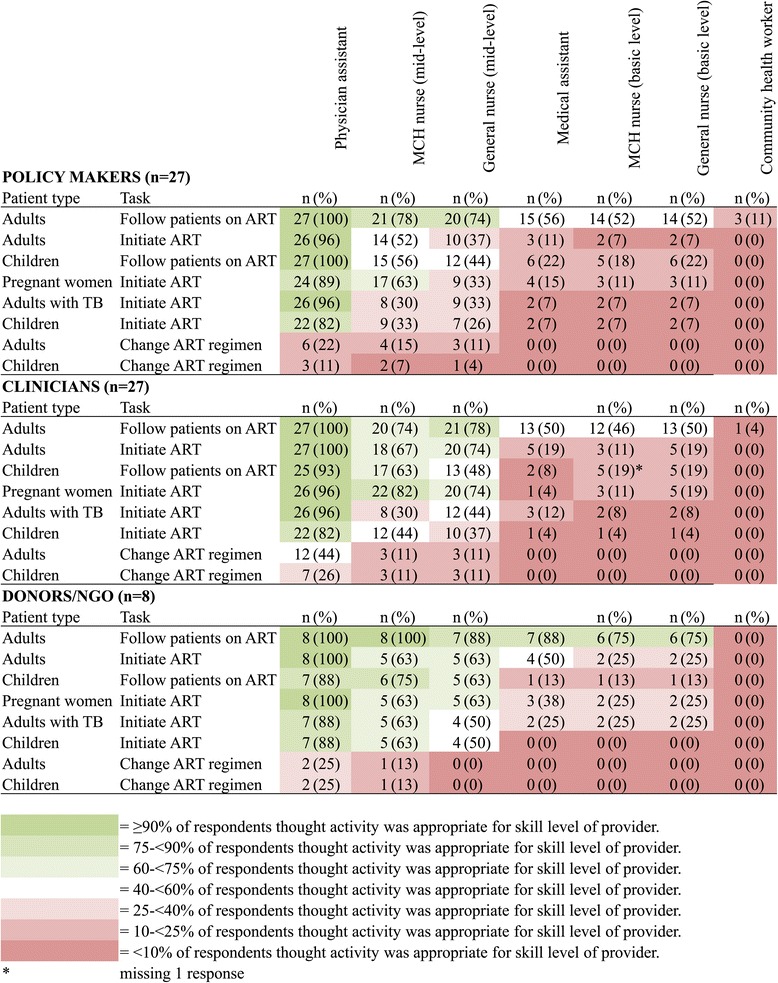
**Support for specific health worker cadres performing ART-related tasks, by interviewee job category (** ***n*** **= 62 interviewees).**

Stratified by current job category, MoH leaders, clinicians, and donors/NGO workers supported the use of NPCs to a similar degree for many combinations of tasks and health worker cadres (Figure 2). They agreed that physician assistants should be able to perform most ART-related tasks and that most complex tasks should not be delegated to any of the seven non-physician cadres. Compared to clinicians, MoH leaders, and to a lesser degree donors/NGO workers, were less likely to support the use of mid-level health workers performing more complex ART-related tasks. For example, 82% of clinicians, but only 63% of MoH leaders and donors/NGO workers, supported the use of mid-level MCH nurses to initiate ART in pregnant women. The majority of clinicians (74%) and donors/NGO workers (63%) supported using general mid-level nurses to initiate ART in adults, while only 37% of MoH leaders shared this perspective.

When respondents were stratified by whether their current job included clinical duties, clinicians (*n* = 28) were more likely than non-clinicians (*n* = 34) to support the use of general mid-level nurses to initiate ART in adults (75% vs. 41%, respectively) and to initiate ART in pregnant women (75% vs. 34, respectively) (not shown in tables). Forty-three percent of clinicians, but only 24% of non-clinicians, thought that physician assistants should be able to change ART regimens in adults. For all other task/health worker combinations, clinicians and non-clinicians agreed within 10 percentage points or less.

### Potential risks of task shifting

Of the 93 open-ended responses that interviewees gave of the risks of using physician assistants to provide ART, the majority related to reduced quality of care (58% of responses), such as poor management of adverse events and improper dosing (Table 3). The next-most-frequently cited risks were insufficient oversight by physicians (12%) and increased drug resistance (11%). Other risks included inadequate technical training of physician assistants (9%), the already overburdened workload of physician assistants (3%), increased patient loss to follow-up (3%), power conflicts with physicians (2%), and insufficient clarity of tasks (1%).

**Table 3:** **Open-ended responses regarding task shifting of ART-related care, among 62 key stakeholders in Mozambique**

	**n (%)**
Potential risks	
Reduced quality of care	53 (58)
Lack of oversight	11 (12)
Drug resistance	10 (11)
Lack of training	8 (9)
High workload of lower cadre health workers	3 (3)
Increased LTFU	3 (3)
Insufficient clarity about tasks	2 (2)
Power conflict with MD	1 (1)
Potential benefits	
Increased ART access	70 (48)
Reduced workload for MDs	25 (17)
Reduced patient waiting time	14 (10)
Improved patient follow-up	10 (7)
Improved quality of ART care	8 (6)
More integrated services	7 (5)
Increased skills health workers	5 (3)
Improved adherence	4 (3)
Reduced HIV stigma	1 (1)
Already occurring	1 (1)
Potential strategies to minimize risks	
Better in-service training	54 (36)
Ongoing supervision and evaluation	53 (36)
Improve communication among clinicians, including clear job descriptions	19 (13)
Job aids	8 (5)
Improve work conditions	4 (3)
Improve patient follow-up	3 (2)
Ensure basic lab tests are done	2 (1)
Improve referral mechanisms	2 (1)
More time for consults	1 (1)
Prioritize OI treatment	1 (1)
Only train good clinicians	1 (1)

### Potential benefits of task shifting

Nearly every respondent cited increased access to ART as the top benefit of task shifting (48% of the 145 responses); reduction of physicians’ workload (17%) and reduction of patients’ waiting times (10%) were the next most commonly cited benefits (Table 3). A few respondents thought that task shifting could lead to closer follow-up of patients (7%), improve the quality of care (6%), and improve integration of HIV services (5%).

### Methods to minimize potential risks and maximize potential benefits of task shifting

Interviewees most commonly stated improved training (36% of 148 responses) and improved supervision (36%) would ensure that non-physicians provide high-quality ART care (Table 3). Improving the communications between health workers—such as clearly defining the tasks of all cadres of health workers, encouraging broad participation in team meetings, and ensuring a positive team dynamic—was a common theme (13%), and improving job aids (5%) and improving working conditions (3%) were mentioned as well.

## Discussion

Respondents agreed that the most highly trained non-physician health workers could perform relatively simple ART-related tasks and that the least trained should not perform ART-related tasks. There was a lower degree of consensus on the delegation of moderately complex ART-related tasks to mid-level cadres of health workers. Clinicians were more supportive of asking general mid-level nurses to initiate in adults and pregnant women than non-clinicians. Respondents commonly cited expanded ART access as the primary advantage of task shifting and reduced quality of care as its primary disadvantage.

Clinicians may have been more supportive of task shifting to lower cadres of health workers than non-clinicians because they are more directly confronted by the reality of constrained access to ART and the complexity of ART provision, or lack thereof; front-line health workers in Mozambique, Zambia [13], and South Africa [12] support task shifting, whereas policy-makers in Uganda have not [15]. Unfortunately, support from MoH leaders is necessary for successful implementation of task shifting [4,10,11]. In the absence of effective leadership, task shifting will likely still occur—as demonstrated in Uganda [15]—but may be more haphazard and therefore pose a greater risk to the quality of care.

The disadvantages of task shifting cited by study respondents—principally, reduced quality of care—differed from those cited by stakeholders in Uganda, which were primarily the cost of training and supervision [15]. Nonetheless, the conclusions of that study mirror our own: that clear guidelines regarding which specific tasks each cadre of health worker can perform are essential to optimize health-care delivery using task shifting.

The interview period for this study (November 2007 to June 2008) overlapped with that of a study assessing the quality of care provided by physician assistants in Mozambique (October to December 2007) [19]. The results of that study raised concerns over the quality of care delivered by physician assistants newly trained in ART provision. Though this study observed that increasing patient volumes were correlated to higher quality care (indicating that as they gained experience, physician assistants would be a viable option to meet the needs of ART-eligible patients), this study temporarily cast doubt over the viability of ART-related task shifting in Mozambique, and consequently, the particular time frame during which interviews were conducted may have coincided with a nadir in support for task shifting among stakeholders in Mozambique. Shortly after this study was conducted, a separate study in Mozambique found that the quality of HIV care provided by physician assistants equaled or exceeded that provided by physicians [6], reaffirming the value of Mozambique’s national programme to train physician assistants to provide ART.

This study has several strengths. To our knowledge, this is the first study to report stakeholder views regarding the delegation of specific tasks in HIV care to specific cadres of health workers. The study instrument included both open- and closed-ended questions, providing more nuanced data. Interviewees included a variety of key stakeholders at the national level, allowing us to characterize the variation in stakeholders’ attitudes by employment category. Interview materials were developed and all interviews were conducted in Portuguese, the native language of study respondents. On the other hand, the study is limited by its relatively small sample size (*n* = 62), though every effort was made to obtain a representative sample of key stakeholders in Mozambique. Also, attitudes around ART-related task shifting may have shifted since interviews were conducted.

Since these interviews were conducted, national-level stakeholders in Mozambique have engaged in continued dialogue around ART-related task shifting. First, in 2008, Mozambique’s MoH adopted a strategic plan for 2008–2015, in part due to the efforts of the working group on human resources chaired by the MoH, which includes 11 in-country donors and partners [20]. One of the working group’s areas of focus is promoting communication between groups of stakeholders—the government, NGO, and donors—to advocate for investment in the full spectrum of human resource development, not only in pre-service training but also increased supervision, support, and mentorship. The main goal of this holistic approach is to promote long-term retention of health workers, but it would address one of the key concerns of stakeholders raised in this study: lack of ongoing support for lower cadre health workers tasked with expanded responsibilities and the reduced quality of care that could result. Given the substantive expansion of task shifting over the last 7 years in Mozambique, this study would likely yield different results if its methods were replicated today. Nonetheless, the snapshot that this study offers is still relevant now for other countries considering the adoption or expansion of task shifting for ART. This study’s results indicate that increased attention should be paid to harmonizing clinician and non-clinician attitudes towards task shifting, as clinicians may initially be more supportive of task shifting than stakeholders without clinical responsibilities. Future research could explore if this dichotomy is observed in other contexts outside Mozambique as well. Second, Mozambique began a concerted effort to increase the number of trained health professionals beginning in 2004 and has made great strides [9]. Though the number of new graduates from health professional programmes has risen, the ratio of health workers per capita in Mozambique is still substantially below the minimum necessary to provide basic health services [2,8].

However, the potential disadvantages of task shifting raised by respondents in our study continue to be concerns in Mozambique, including reduced quality of care, inadequate training of health workers, and lack of ongoing support for lower cadre health workers as they assume new duties [13,20]. These issues are exacerbated by the poor working conditions that all health workers face in Mozambique due to a variety of factors, such as overburdened workloads, inability to take time off for continuing education, and inadequate infrastructure and equipment [13]. Currently, there are 590 000 adults and 100 000 children in Mozambique who are in need of ART but unable to access treatment [1], in part due to the severe shortage of trained health professionals. Coordination, communication, and strong commitment from the MoH and other national stakeholders will continue to be critical to close this treatment gap and strengthen the health system for all care providers in Mozambique.

In the future, the tension between the political imperative to expand ART access and concerns about the quality of resulting care will no doubt persist, which will continue to drive task shifting policies and strategies. For example, at the time of this research, mid-level nurses were not considered capable of providing ART, yet today many provide Option B+ to pregnant and nursing women. Addressing concerns about how to maintain high-quality care while reaching all eligible patients will require adequate training, ongoing support, and rigorous monitoring of future efforts to shift ART provision to lower cadres of health workers.

Further research is needed to investigate why the dichotomy in opinion between clinicians and non-clinicians exists and whether it persists and to define the elements of successful leadership and governance necessary to implement task shifting for HIV/AIDS care and in general. The knowledge gained from studying the process of formulating clear policies around ART-related task shifting could provide a blueprint for future efforts to shift tasks to lower cadres health workers, where appropriate, such as the provision of ART through Option B+ to pregnant women and infants.

## Conclusions

National stakeholders in Mozambique generally agree on whether specific health worker cadres should or should not perform the most and least complex ART-related tasks. However, clinicians are more likely to support delegating moderate-complexity tasks to mid-level health workers than non-clinicians. Providing adequate training, supervision, and improving general health worker conditions may alleviate concerns about the quality of care under task shifting. Harmonizing attitudes towards task shifting among key stakeholders and achieving consensus regarding which specific ART-related tasks to delegate to lower cadres of health workers will facilitate successful implementation of policies to scale up ART-related task shifting.

## Additional file

Additional file 1:
**Questionnaire for Policy Makers on the Role of Mid-level Providers in ART Provision.** This is the English translation of the Portuguese study instrument that was used to conduct all interviews.

## Abbreviations

ART: Antiretroviral therapy
MoH: Ministry of Health
NGO: Non-governmental organization
WHO: World Health Organization
