# Supplementary material for: Perspectives of key stakeholders regarding task shifting of care for HIV patients in Mozambique: a qualitative interview-based study with Ministry of Health leaders, clinicians, and donors
Source: Hum Resour Health. 2015 Apr 1;13:18. doi: 10.1186/s12960-015-0009-3 (PMC4387582; doi:10.1186/s12960-015-0009-3)
Supplement: Additional file 1: — Questionnaire for Policy Makers on the Role of Mid-level Providers in ART Provision. This is the English translation of the Portuguese study instrument that was used to conduct all interviews. [file 12960_2015_9_MOESM1_ESM.doc]

**Questionnaire for Policy Makers on the Role of Mid-level Providers in ART Provision**

**I. Background**

1. Date ____/____/______
2. Name: ________________________________________________________
3. Sex: M/F
4. Date of Birth: _____/_____/__________
5. Training:
   1. Physician
   2. Specialized Physician (specialty)________________
   3. Medical Officer
   4. Other (Specify) ________________________
6. When did you finish your initial training (month/year)_______/___________
7. When did you finish your post graduate training (month/year)_______/___________
8. Current Position: _____________________________________________

**III. Experience working in the national health service**

1. Experience working in the national health service (list chronologically your location of work, function, and length of time in the position).

| **Location (health facility, city, province)** | **Position** | **Length of time in the position** |
| --- | --- | --- |
|  |  |  |
|  |  |  |
|  |  |  |
|  |  |  |
|  |  |  |
|  |  |  |
|  |  |  |

**IV. Impressions about human resources in ART expansion**

1. What are your impressions about the current initiative to use physician assistants to provide ART in adults?
   1. Favorable
   2. Not favorable
   3. Neutral

**Explain your response:**

1. Since being authorized to prescribe ART, has the role of medical officers changed at the level of the HIV clinic? Yes/No

If yes, how?

1. Do you think that the role of medical officer should change due to the HIV epidemic? Yes/No
2. What are your impressions about the current initiative to use medical officers to prescribe ART in children?
   1. Favorable
   2. Not favorable
   3. Neutral

**Explain your response:**

1. What are your impressions about the current initiative to use medical officers to prescribe ART in pregnant women?
   1. Favorable
   2. Not favorable
   3. Neutral

**Explain your response:**

1. What are your impressions about the current initiative to use medical officers to prescribe ART in patients with tuberculosis?
   1. Favorable
   2. Not favorable
   3. Neutral

**Explain your response:**

1. Limitations of activities by level of health worker:

|  | Medical Officer | Mid-level Nurse | Mid-level MCH Nurse | Basic-level Nurse | Basic-level MCH Nurse | Medical auxiliaries | Community Health Workers |
| --- | --- | --- | --- | --- | --- | --- | --- |
| Staging, triage and patient referral | Yes/No | Yes/No | Yes/No | Yes/No | Yes/No | Yes/No | Yes/No |
| Preparation of patients to initiate ART | Yes/No | Yes/No | Yes/No | Yes/No | Yes/No | Yes/No | Yes/No |
| Follow-up of patients in ART without complications | Yes/No | Yes/No | Yes/No | Yes/No | Yes/No | Yes/No | Yes/No |
| Initiation of ART for adult patients without complications (those taking the first line) | Yes/No | Yes/No | Yes/No | Yes/No | Yes/No | Yes/No | Yes/No |
| Initiation of ART in pregnant women | Yes/No | Yes/No | Yes/No | Yes/No | Yes/No | Yes/No | Yes/No |
| Follow-up of children <15 in ART | Yes/No | Yes/No | Yes/No | Yes/No | Yes/No | Yes/No | Yes/No |
| Initiation of ART in children in ART | Yes/No | Yes/No | Yes/No | Yes/No | Yes/No | Yes/No | Yes/No |
| Initiation of ART in patients with TB | Yes/No | Yes/No | Yes/No | Yes/No | Yes/No | Yes/No | Yes/No |
| Change of first line ARVs to alternative lines in adults | Yes/No | Yes/No | Yes/No | Yes/No | Yes/No | Yes/No | Yes/No |
| Change of first line ARVs to alternative lines in children | Yes/No | Yes/No | Yes/No | Yes/No | Yes/No | Yes/No | Yes/No |
| They should have the same responsibilities as physicians trained in ART (substitute physicians with medical officers for all categories beyond medical officer) | Yes/No | Yes/No | Yes/No | Yes/No | Yes/No | Yes/No | Yes/No |
| At what level should they be authorized to provide ART: | Yes/No | Yes/No | Yes/No | Yes/No | Yes/No | Yes/No | Yes/No |
| - Central/Provincial/General Hospital | Yes/No | Yes/No | Yes/No | Yes/No | Yes/No | Yes/No | Yes/No |
| - Rural Hospital | Yes/No | Yes/No | Yes/No | Yes/No | Yes/No | Yes/No | Yes/No |
| - Urban Health Center | Yes/No | Yes/No | Yes/No | Yes/No | Yes/No | Yes/No | Yes/No |
| - Rural Health Center | Yes/No | Yes/No | Yes/No | Yes/No | Yes/No | Yes/No | Yes/No |
| Leadership/management of ART activities | Yes/No | Yes/No | Yes/No | Yes/No | Yes/No | Yes/No | Yes/No |

1. In your opinion, what are the greatest risks for using non-physicians in expanding ART? List 3 in order of importance:

1.

2.

3.

1. And the greatest benefits? List 3 in order of importance:

1.

2.

3.

1. What are the most important steps to ensure quality of ART services by non-physicians? List 3 in order of importance

1.

2.

3.
